# Towards a Continuous Manufacturing Process of Protein-Loaded Polymeric Nanoparticle Powders

**DOI:** 10.1208/s12249-020-01814-w

**Published:** 2020-10-06

**Authors:** Stefan Schiller, Andrea Hanefeld, Marc Schneider, Claus-Michael Lehr

**Affiliations:** 1 Department of Pharmacy, Biopharmaceutics & Pharmaceutical Technology, Saarland University, Saarbrücken, Germany; 2 Department of Pharmaceutical Technologies, Merck Healthcare KGaA, HPC D039/002, Frankfurter Str. 250, 64293 Darmstadt, Germany; 3 Global Healthcare Operations Innovation Network, Merck Healthcare KGaA, Darmstadt, Germany; 4 Helmholtz Institute for Pharmaceutical Research Saarland (HIPS), Helmholtz Center for Infection Research (HZI), Saarland University, Saarbrücken, Germany

**Keywords:** continuous manufacturing, PLGA nanoparticles, focused ultrasound, spray drying, protein delivery

## Abstract

To develop a scalable and efficient process suitable for the continuous manufacturing of poly(lactic-co-glycolic acid) (PLGA) nanoparticles containing ovalbumin as the model protein. PLGA nanoparticles were prepared using a double emulsification spray-drying method. Emulsions were prepared using a focused ultrasound transducer equipped with a flow cell. Either poly(vinyl alcohol) (PVA) or poloxamer 407 (P-407) was used as a stabilizer. Aliquots of the emulsions were blended with different matrix excipients and spray dried, and the yield and size of the resuspended nanoparticles was determined and compared against solvent displacement. Nanoparticle sizes of spray-dried PLGA/PVA emulsions were independent of the matrix excipient and comparable with sizes from the solvent displacement method. The yield of the resuspended nanoparticles was highest for emulsions containing trehalose and leucine (79%). Spray drying of PLGA/P-407 emulsions led to agglomerated nanoparticles independent of the matrix excipient. PLGA/P-407 nanoparticles pre-formed by solvent displacement could be spray dried with limited agglomeration when PVA was added as an additional stabilizer. A comparably high and economically interesting nanoparticle yield could be achieved with a process suitable for continuous manufacturing. Further studies are needed to understand the robustness of a continuous process at commercial scale.

## INTRODUCTION

A major challenge in the translation and commercialization of nanomaterials in medicine is the development of adequate pharmaceutical production processes that work equally well at large scale as at lab scale as (1,2). While several marketed pharmaceutical products employ nanotechnology, very little is publicly known about production processes and the translation from research to commercial scale (3). Continuous processes are often investigated (2,4,5), as they are considered easy to scale and more efficient, allow for simple process monitoring, and typically lead to less batch-to-batch variation. Although continuous nanoparticle precipitation methods—sometimes called flash nanoprecipitation—exist (5–7), in general, they are not suitable to produce nanoparticles of hydrophobic polymers and hydrophilic cargos. Such systems are often produced using the double emulsion method (8): the hydrophilic drug is dissolved in water and emulsified into a non- or partially miscible solvent containing the polymer. This two-phase system is further emulsified into an outer aqueous phase containing a stabilizer. The solvent is subsequently removed and the polymer precipitates to form nanoparticles around the hydrophilic drug. The critical steps of emulsification and solvent removal are typically done with batch processing (9). We previously reported the use of a focused ultrasound transducer coupled with a flow-through cell for a contact-free and scalable emulsification capable of continuous processing (9). The logical next step would be to also adapt the solvent evaporation to a continuous process, for example, by spray drying.

Spray drying is a continuous, fast, and efficient process for solvent removal. It may be used to transform preformed nanoparticle suspensions to dry powders commonly known as “Trojan particles,” nanoembedded microparticles, or nanoparticles-in-microparticles (10,11). The direct precipitation and drying of nanoparticles has been described as the emulsion spray drying approach, using single water/oil (W/O) emulsions (12–15). The size of the precipitated nanoparticles is controlled by the atomization of the feed rather than by the droplet size of the dispersed phase of the emulsion.

However, the generation of submicron droplets and the collection of submicron particles with sufficient yield are very challenging with conventional equipment, and the throughput of available specialized equipment is severely limited compared with conventional nozzles, cyclones, and filter bags (16).

Spray dying of water/oil/water (W/O/W) double emulsions allows for the *in situ* generation of polymeric nanoparticles with hydrophilic cargo while simultaneously embedding them in a stabilizing powder matrix. The concept has been described for batch mode emulsification and conventional spray drying equipment (17,18), but little is known about the factors influencing nanoparticle size and yield. The aim of this study was to investigate whether poly(lactic-co-glycolic acid) (PLGA) nanoparticles can be produced with acceptable yield and particle size distribution by the double emulsion method using scalable methods fit for continuous manufacturing. Different emulsion stabilizers and matrix components were investigated for their influence on nanoparticle size and yield.

## MATERIALS AND METHODS

### Materials

Poly(lactic-co-glycolic acid) (PLGA, Resomer® RG 503 H) was purchased from Evonik Industries (Essen, Germany). Poloxamer 407 (P-407), Kollidon® 30 (K30), and Kollidon® VA64 (VA64) were kindly provided by BASF (Ludwigshafen, Germany). Poly(vinyl alcohol) (PVA; Mowiol® 4–88) was obtained from Kuraray Europe GmbH (Hattersheim am Main, Germany). Ovalbumin grade V, as well as all other chemicals, was obtained from Merck KGaA (Darmstadt, Germany). Water was purified with a Milli-Q® system (Merck KGaA; Darmstadt, Germany).

### Emulsion Preparation

All solutions were freshly prepared and filtered through 0.2-μm membrane filters (polytetrafluoroethylene for organic solutions or polyethersulfone for aqueous solutions). Double emulsions were prepared using focused ultrasound according to a previously described method (9). In brief, PLGA was dissolved to 12 mg/mL in 70 mL ethyl acetate and added to 14 mL of an aqueous solution of 6 mg/mL ovalbumin. The mixture was circulated through the flow cell of a commercial focused ultrasound transducer (Covaris S2x, Covaris Inc.; Woburn, MA, USA) using a peristaltic pump at 50 mL/min. The mixture was homogenized for 5 min at intensity 10, duty factor 50%, 300 cycles per burst. An aqueous solution, 140 mL of 20 mg/mL PVA or P‐407, was added, and the mixture was again emulsified by focused ultrasound for 45 min (same settings). An aliquot of this emulsion was precipitated by solvent displacement (10‐fold dilution with purified water). The particle size distribution of the obtained nanoparticle suspension was measured by dynamic light scattering as described below. The results served as a control for in‐use emulsion stability and as a target for nanoparticles precipitated by spray drying.

### Spray Drying

Aliquots of the double emulsion, consisting of dry mass ratios of 1 part PLGA and 3.3 parts PVA or P-407, were either directly spray dried or spiked with 9 parts matrix excipient. The aliquots were fed at 2 mL/min into a ProCepT 4M8-TriX lab scale spray dryer equipped with a straight and a conical drying column and a small cyclone (ProCepT nv; Zelzate, Belgium); atomized with a 1.0 mm bi-fluid nozzle and 10 L/min nitrogen; and dried at 80°C inlet drying air temperature, 0.4 m^3^/min drying air flow, 40–42°C cyclone outlet temperature, and a pressure drop of 60–65 mbar over the cyclone. Ambient conditions were between 20 and 23°C and 40–60% rh.

### Particle Size and Yield Determination

Spray-dried powders were reconstituted with purified water at approximately 0.5 mg/mL PLGA by shaking for 30–60 min and passed through a 1.0 μm glass microfiber syringe filter to remove agglomerated PLGA particles and incompletely dissolved matrix excipients. The filtered particle suspensions were analyzed by dynamic light scattering (Zetasizer Nano ZS, Malvern Instruments; Worcestershire, UK; *λ* = 633 nm, 25.0 ± 0.1°C, backscatter mode, cumulants fit).

Filtered and unfiltered suspensions were centrifuged for 5 min at 21,000x*g* to collect the PLGA particles. The pellet was washed twice with water, dried in a rotary vacuum concentrator (RVC 2–33 IR, Martin Christ**;** Osterode, Germany), and subsequently weighed. The nanoparticle yield was calculated by dividing the mass of the pellet from the filtered suspension by the mass of the pellet from the corresponding unfiltered suspension.

## RESULTS AND DISCUSSION

Double emulsions containing PLGA and either PVA or P-407 as the nanoparticle stabilizer were spray dried and analyzed for changes in particle size distribution and the resulting nanoparticle yield. The emulsions were either sprayed “as is” or after the addition of matrix excipients: trehalose and mannitol as desiccoprotectants, leucine to increase the dispersibility of the spray-dried powders (19)); VA64 and K30 due to their ability to sterically stabilize drug nanoparticles (20); or the further addition of the nanoparticle stabilizers PVA and P-407 also as matrix excipients. Nanoparticle precipitation from PLGA/PVA or PLGA/P-407 double emulsions by solvent displacement without any additives or spray drying served as the control.

**Fig. 1:**
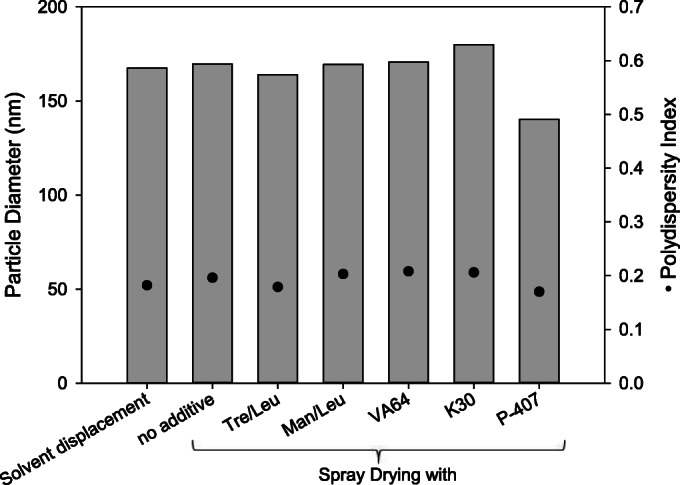
Resulting nanoparticle sizes after spray drying of PLGA/PVA double emulsions with and without the addition of different matrix excipients. Spray-dried powders were reconstituted in purified water, then aggregates were removed by filtration, and particle size distribution was measured by dynamic light scattering. Nanoparticles precipitated by solvent displacement without spray drying served as the control. Measurements were done in triplicate, RSD < 0.5%

Spray drying of double emulsions containing PLGA/PVA yielded nanoparticles of similar size as precipitation by direct solvent displacement (Fig. 1). The addition of different matrix excipients to the emulsions before drying did not influence the resulting nanoparticle size. Only P-407 seemed to decrease the resulting nanoparticle size. P-407 is a surface-active block copolymer suitable for the stabilization of PLGA double emulsions and suspensions alone, resulting in particles of a smaller hydrodynamic diameter when using the same manufacturing method and parameters (9). A possible explanation is that P-407 may replace PVA, to a certain extent, at the liquid/liquid interface before drying or at the particle/liquid interface after drying and reconstitution.

**Fig. 2:**
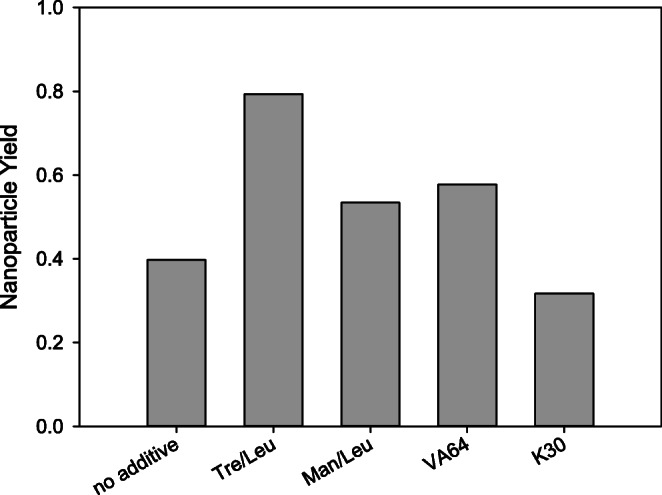
Resulting nanoparticle yield after spray drying of PLGA/PVA double emulsions with and without the addition of different matrix excipients. Nanoparticles were isolated from the matrix after reconstitution of the complete batch in purified water, then aggregates were removed by filtration, and the mass was determined after drying

In contrast to the mean particle size, the nanoparticle mass recovery from spray-dried powders is influenced by the choice of matrix (Fig. 2). Spray drying of the emulsion with no further addition of excipients resulted in a nanoparticle yield of 40%. Adding K30 to the emulsion is not beneficial for the nanoparticle yield. Adding mannitol/leucine or VA64 moderately increases the yield to 50–60%. The best result of 79% was obtained when trehalose/leucine were added to the emulsion. A nanoparticle yield of 79% is considerably higher than previously reported for the continuous production of PLGA nanoparticles using focused ultrasound followed by solvent displacement (9). The reported yield may already be economically acceptable when reproduced on a commercial scale.

After the proposed process, excess stabilizer and free drug would remain in the spray-dried powder. The spray-dried powder could be directly used for further downstream processing (*e.g.*, tableting) into a final dosage form for applications where free drug and stabilizer are not an issue. Alternatively, the dry powder intermediate may be purified before further processing. Centrifuge purification works well for PVA and is well established at lab scale, but the scalability is limited and particles may deform or agglomerate under stress (9). Continuous purification methods, such as cross-flow filtration, are preferred at larger scale, but are challenging for formulations containing PVA: Residual amounts of PVA were found to be several times higher after filtration than after centrifugation (21,22). P-407 has been previously shown to work well with the double emulsion solvent displacement method and is easily removed by crossflow filtration (9). Therefore, we evaluated the potential of using P-407 with the double emulsion spray drying method.

**Fig. 3:**
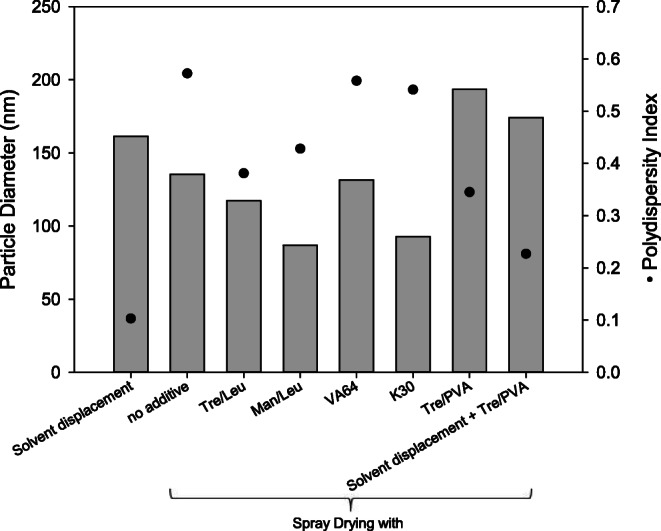
Resulting nanoparticle sizes after spray drying of PLGA/P-407 double emulsions with and without the addition of different matrix excipients. Spray-dried powders were reconstituted in purified water, then aggregates were removed by filtration, and particle size distribution was measured by dynamic light scattering. Nanoparticles precipitated by solvent displacement without spray drying served as the control. Measurements were done in triplicate, RSD < 0.5%

Particles of PLGA/P-407 precipitated from the emulsion by solvent displacement instead of spray drying had a very narrow size distribution (indicated by a polydispersity index [PDI] of 0.10) and served as the control. Spray drying of emulsions stabilized with P-407 yielded agglomerated particles (Fig. 3): Spray drying without further additives resulted in a PDI of 0.57. The addition of VA64 and K30 did not improve the particle size distribution (PDI: 0.54–0.56). Using trehalose and leucine or mannitol and leucine as the matrix excipients resulted in a lower but still unsatisfactory PDI of 0.38–0.43.

**Fig. 4:**
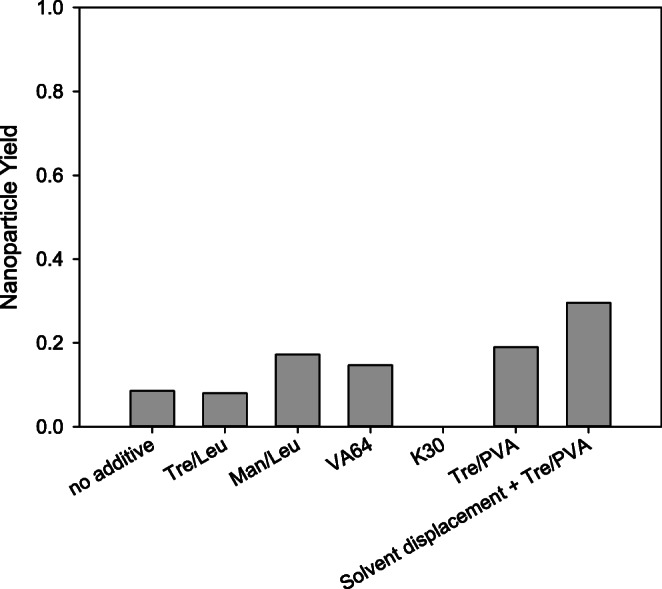
Resulting nanoparticle yield after spray drying of PLGA/P-407 double emulsions with and without the addition of different matrix excipients. Nanoparticles were isolated from the matrix after reconstitution of the complete batch in purified water, then aggregates were removed by filtration, and the mass was determined after drying. The nanoparticle yield with K30 was too low to determine by weighing and, as such, is reported as 0

As PLGA/PVA emulsions could be successfully spray dried when spiked with P-407, it was tested whether the addition of PVA and trehalose to a PLGA/P-407 emulsion could positively influence nanoparticle stability. The resulting PDI of 0.35 was only slightly better than when using trehalose and leucine. This indicates that the presence of PVA is already needed during the emulsion step to exert its stabilizing effect during spray drying. An acceptable PDI of 0.23 could only be achieved when spray drying a suspension previously precipitated by solvent displacement after the addition of PVA and trehalose. However, the nanoparticle yield was unacceptable for all tested formulations of PLGA/P-407 particles (Fig. 4) and was considerably lower than previously reported for focused ultrasound followed by solvent displacement (9). The superiority of PVA may be attributed to the formation of a thicker and denser surface layer (indicated by the larger resulting hydrodynamic particle sizes) and stronger surface binding and retention (23,24), whereas P-407 adsorbs weaker with its middle poly(oxypropylene) block (25).

## CONCLUSION

An economically interesting nanoparticle yield can be achieved using continuous manufacturing and drying methods. PVA stabilizes emulsion droplets and PLGA nanoparticles during spray drying to achieve a high nanoparticle yield and good particle size distribution, especially in the presence of trehalose and leucine. P-407 is not an effective stabilizer for the double emulsion spray drying method, despite being effective for solvent displacement. Further development work is needed to link the individual processes in a continuous line and to investigate process robustness, scalability, and protein loading efficiency. Further optimization of the emulsion step or use of different emerging emulsion techniques, such as microfluidics, would allow for completely continuous manufacturing of polymeric nanoparticles with an acceptable yield for commercial-scale manufacturing.
